# Mechanosensing in the Physiology and Pathology of the Gastrointestinal Tract

**DOI:** 10.3390/ijms24010177

**Published:** 2022-12-22

**Authors:** Job Baffin Kola, Tibor Docsa, Karen Uray

**Affiliations:** 1 Department of Medical Chemistry, Faculty of Medicine, University of Debrecen, 4032 Debrecen, Hungary; 2 Center of Excellence, The Hungarian Academy of Sciences, 4032 Debrecen, Hungary

**Keywords:** mechanotransduction, gastrointestinal, ileus, irritable bowel disease

## Abstract

Normal gastrointestinal function relies on sensing and transducing mechanical signals into changes in intracellular signaling pathways. Both specialized mechanosensing cells, such as certain enterochromaffin cells and enteric neurons, and non-specialized cells, such as smooth muscle cells, interstitial cells of Cajal, and resident macrophages, participate in physiological and pathological responses to mechanical signals in the gastrointestinal tract. We review the role of mechanosensors in the different cell types of the gastrointestinal tract. Then, we provide several examples of the role of mechanotransduction in normal physiology. These examples highlight the fact that, although these responses to mechanical signals have been known for decades, the mechanosensors involved in these responses to mechanical signals are largely unknown. Finally, we discuss several diseases involving the overstimulation or dysregulation of mechanotransductive pathways. Understanding these pathways and identifying the mechanosensors involved in these diseases may facilitate the identification of new drug targets to effectively treat these diseases.

## 1. Introduction

The ultimate goal of the gastrointestinal (GI) tract is to provide nutrients and water to the organism, followed by the elimination of wastes. Thus, the regulation of intestinal motility is centered on optimizing nutrient delivery, and the ability to sense and respond to mechanical forces is crucial in optimizing the movement of ingested material through the GI tract. Many chemical sensors participate in the regulation of gastrointestinal motility. In addition to chemical signaling, normal GI function is dependent on mechanotransduction. The gut must sense a complex range of passive and active mechanical forces along the gastrointestinal tract and transduce those signals to chemical signals to respond appropriately (see Ref. [1] for a complete review of mechanical forces in the gut). While some cell types in the gut are specialized mechanosensors, virtually all gastrointestinal cell types, including epithelial cells, smooth muscle cells, interstitial cells of Cajal, enteric neuronal cells, and even immune cell types, can respond to mechanical forces to participate in the coordinated movement of ingested material through the gastrointestinal tract. As the gut is a mechanically active organ, these mechanosensitive cells must discriminate between normal mechanical activity and mechanical signals. While mechanotransduction in the gut has been recognized since the late 1800s, e.g., Bayliss and Starling’s “law of the intestine”, the identification of mechanosensors that mediate mechanotransduction is more recent [2]. In addition to the role of mechanotransduction in normal physiology, the dysregulation of mechanotransduction plays a role in the pathophysiology of the gut. Both aberrant mechanosignaling and the overstimulation of mechanosensitive pathways, e.g., gut distension in obstructive bowel disease, can lead to pathological outcomes. We briefly review normal mechanosensing in the gut, focusing on the role of mechanotransduction in regulating GI motility. Then, we discuss the dysregulation of mechanotransduction mechanisms leading to GI motility disorders.

## 2. The Role of Mechanotransduction in Normal Gastrointestinal Physiology

The gut must sense mechanical forces to coordinate the movement and absorption of ingested material along the GI tract. Mechanical signals, such as shear stress (changes in luminal flow), compression (due to GI contractions), and stretch (due to luminal distension), are sensed by cells throughout the GI tract to coordinate GI motility. In addition, the gut must sense the mechanical properties, amount, and location of ingested material along the GI tract to properly regulate the absorption of nutrients, storage of ingested materials, and defecation [3]. Mechanosensors convert mechanical signals into chemical signals. Mechanosensation relies on both specialized sensory cells, including enterochromaffin cells and mechanosensory neurons, and non-specialized cells, such as smooth muscle cells and interstitial cells of Cajal (ICCs) [4]. We describe below the contribution of each cell type to sensing mechanical signals. The molecules involved in mechanotransduction are reviewed in Ref. [5], and we only highlight a few mechanosensitive molecules, which are mostly ion channels, below. Table 1 shows the potential primary mechanosensors in the GI tract.

### 2.1. Mechanosensitive Cells

#### 2.1.1. Enterochromaffin Cells

Enterochromaffin cells (ECs), which are distributed along the entire length of the GI tract, are the most abundant epithelial enteroendocrine cells in the gut mucosa. These specialized mechanosensitive cells release serotonin in response to mechanical stimulation, including shear stress, stretch/distension, and compression [22,23,24,25]. In fact, most of the body’s serotonin is synthesized and stored in the ECs [26]. Serotonin secretion activates the intrinsic and extrinsic primary afferent neurons to coordinate the peristalsis, vasodilation, and propulsion of the intestinal contents [27,28]. 

The exact mechanism(s) of mechanosensitivity in ECs is still under investigation. EC cells are sparse and difficult to culture, making specific investigations in the GI tract difficult [29]. The basic signaling pathway involves the release of ATP and UTP from cells in response to mechanical signals. ATP and UTP transduce mechanosensory signals via purinergic (P2Y) receptors in the large intestine or colon (other signal transducers may be involved in other parts of the gastrointestinal tract), resulting in the release of serotonin [30]. Serotonin then regulates peristalsis and secretion in the gut by stimulating enteric neurons [31]. Disruption of caveolae prevents serotonin release, indicating that caveolin-mediated scaffolding is necessary for the signal transduction pathway [25]. 

The Piezo2 channels have emerged as a primary candidate for mechanosensing on the luminal side of the GI tract via EC cells [6,7]. In an EC cell model, Wang et al. showed that pressure results in the release of serotonin, which is prevented by the siRNA knockdown of Piezo2, although these experiments were performed in QGP-1 cells, which are pancreatic islet carcinoma cells, not intestinal EC cells [6]. Alaino et al. showed that Piezo2 is expressed in EC cells close to serotonin vesicles [7]. Piezo2 activation results in Ca^2+^ influx to trigger downstream signaling. However, Piezo2 channels inactivate in a significantly shorter time (10 s) than the serotonin release. Thus, other pathways may be involved in amplifying or prolonging the signal transduction pathway resulting in serotonin release [23,32]. In addition to sensing distension, a population of enteroendocrine cells (EECs) expressing Piezo2 conveys the ability to alter both small and large intestinal motility in response to the physical properties of the luminal contents [8]. 

ECs play an important role in regulating intestinal motility in response to changes in luminal contents. Inhibition of serotonin synthesis in EC cells causes abnormal colonic motility [28]. However, colonic motility is largely intact after removing the mucosa; therefore, other cell types also mediate mechanotransduction pathways to regulate gastrointestinal motility. 

#### 2.1.2. Enteric Neurons

The extensive enteric nervous system (ENS) is spread throughout the gastrointestinal tract, with submucosal and myenteric plexi between the mucosal and smooth muscle layers and between the two smooth muscle layers, respectively, of the gastrointestinal tract. The ENS includes mechanosensitive neurons that regulate GI motility autonomously from the central nervous system. In animal models, 15–45% of myenteric neurons in the small intestinal and colon are mechanosensitive [33,34,35]. Stimulation of mechanosensitive neurons, but not all neurons of the myenteric plexus, causes active spike discharges [36]. Sustained stretch elicits tonic spike discharge, while brief mechanical stimulation elicits a phasic spike discharge, indicating that mechanosensitive enteric neurons respond differently to different mechanical stimuli; therefore, different mechanisms may be active in response to different stimuli [37,38]. The compression or stretch of mechanosensitive motor neurons elicit changes in intestinal motility. However, enteric neurons are not responsive to shear stress [39,40]. Muscle tone can also act as a co-initiator of peristalsis in addition to contraction [41].

Expression of transient receptor potential (TRP)A1 channels is enriched in the nerve endings of sensory neurons in the gastrointestinal tract and may function as mechanosensors [9]. TRPV4 channels are also expressed in colonic afferents and may play a role in mechanosensing [14]. Piezo1 channels are expressed in both the body and fibers of myenteric and submucosal mechanosensitive neurons; however, pharmacologic inhibition of Piezo1 did not alter mechanotransduction [42]. Thus, the role of Piezo1 in mechanotransduction is unclear. Furthermore, the role of serotonin in distension-evoked peristalsis is unclear. Inhibition of mucosal tryptophan hydroxylase-1, which catalyzes the rate-limiting step in serotonin synthesis, did not affect gastrointestinal transit [43,44]. Other mechanosensitive channels, such as large-conductance-voltage- and Ca^2+^-activated K^+^ channels (BKCa channels), as well as two-pore domain K^+^ (K2P) channels, may be involved in mechanotransduction in enteric neurons; however, the mechanism of mechanotransduction in enteric neurons is still unclear. 

#### 2.1.3. Interstitial Cells of Cajal

ICCs are interposed between enteric nerves (myenteric region) and GI smooth muscle cells. In addition to the pacemaker function of ICCs (i.e., slow waves), these cells are implicated in myogenic responses. Sustained distension of ICCs may increase the amplitude of slow waves [45]. Notably, transgenic mice without intramuscular ICCs do not respond to stretch in the stomach, indicating a function for ICC in stretch-dependent responses in the gastrointestinal tract [46]. Eicosanoids may mediate this stretch response in ICCs. Mechanical regulation of ICC modulates the electrical slow wave, resulting in membrane potential that initiates GI contraction [15,47]. L-type Ca channels and sodium voltage-gated channel alpha subunit 5 (SCN5A) channels have been implicated in the mechanosensitivity of ICCs [48]. Strege et al. showed that stretch increased slow-wave frequency via SCN5A [17].

#### 2.1.4. Smooth Muscle Cells 

Smooth muscle cells (SMCs), in contrast to enteric neurons and enterochromaffin cells, are non-specialized mechanosensitive cells that alter GI function in response to stretch. Smooth muscle cells serve as the motor function’s final effectors and have the peculiar ability to contract independently of the neuronal input (myogenic reflex) [49]. Ion channels are likely mechanosensors, given their location in the cell membrane and their ability to regulate membrane depolarization and subsequent Ca^2+^ fluxes. Thus, most research concerning mechanotransduction in smooth muscle has focused on ion channels. Several mechanosensitive ion channels can regulate smooth muscle function, including non-selective cation channels, L-type Ca^2+^ channels, BK channels, and TRPC4 and TRPC6 channels [12,50,51,52]. Deletion of TRPC4 and TRPC6 in gastrointestinal smooth muscle cells impairs intestinal motility, supporting a role for these ion channels in smooth muscle myogenic response [53]. Calcium channels are activated in response to positive pressure in jejunal smooth muscle cells [18]. Although several mechanosensitive ion channels have been implicated in intestinal and colonic smooth muscle responses to mechanical signals, the specific signal transduction pathways are poorly understood.

#### 2.1.5. Macrophages

Intestinal macrophages reside in the muscle layers of the gut and interact with other cell types, including smooth muscle cells, enteric neurons, and ICCs [54]. Resident muscularis macrophages can be activated by mechanical forces, such as those experienced during trauma or exploratory laparotomy [55,56,57]. Mechanical strain increases the expression of inflammatory mediators, including interleukin (IL)-6, IL-1β, macrophage inflammatory protein (MIP)-1α, and MIP-2 [58]. Although little is known about mechanosensors in intestinal macrophages, mechanosensation has been detected in other macrophages. In THP-1 cells (a monocyte cell line), cyclic stretch elicits IL-8 secretion [59]. Cyclic stretch of peripheral blood mononuclear cells changes macrophage polarization [60]. These studies clearly demonstrate the mechanosensitivity of macrophages; however, the mechanosensors in macrophages are not well understood. In bone marrow-derived macrophages, Piezo1 channels mediate inflammatory mediator expression in response to changes in matrix stiffness [61]. TRPV4 channels modulate macrophage phenotype in response to matrix stiffness [21]. Cell–cell and cell–matrix adhesions also likely mediate phenotypic changes in macrophages in response to stretch [62].

## 3. Examples of Mechanical Signals in the Regulation of Gastrointestinal Motility

Coordinated responses to mechanical signals, in conjunction with chemical and neural signals, are essential for normal gastrointestinal motility. The myogenic response of the gastrointestinal smooth muscle layers, which play a role in virtually all motor activities in the gut, has been known for nearly 75 years, and much of the sympathetic circuitry and chemical signals have been elucidated over the past few decades. However, despite the well-known role that mechanical signals play in coordinating gastrointestinal motility, the molecular mechanisms for mechanotransduction are poorly understood. The mechanical responses involve multiple levels of complex responses across multiple organs to coordinate intestinal motility and optimize nutrient absorption. We describe a few responses to mechanical signals in the gastrointestinal tract to highlight our poor understanding of the mechanosensitive signal transduction pathways. Given the complexity of the responses, we will focus on the mechanosensing functions. Based on these few examples of the role of mechanotransduction in the gastrointestinal tract, it becomes clear that although the neural circuitry has been elucidated, the molecular mechanisms (i.e., identification of the mechanosensitive molecules) of mechanosensing are still not well understood. 

### 3.1. Adaptive Relaxation of the Stomach 

Adaptive relaxation of the stomach is the reflexive relaxation in response to distension of the stomach. Increased volume in the stomach triggers tonic relaxation in the gastric fundus. This reflex involves extrinsic and enteric nerves. The extrinsic neural response involves a vago–vagal reflex in response to stretch receptors, which involve possible mechanosensitive sympathetic neurons terminating in the wall of the stomach, resulting in the release of nitric oxide and the relaxation of the stomach muscle. However, adaptive relaxation can also occur in the absence of extrinsic innervation, indicating that intrinsic mechanisms contribute to adaptive relaxation. Mechanoreceptors in the wall of the proximal stomach can sense gastric distension. G cells, a specialized population of enteroendocrine cells that produce gastrin, are activated in response to the distension of the stomach, even in the absence of extrinsic innervation [63]. However, the mechanism of G cell activation is unclear. Although intramuscular ICCs may be involved in the distension-induced regulation of myogenic tone, they are not required for adaptive relaxation [64]. 

The actual mechanosensing molecules involved in adaptive relaxation are not known. Piezo1 channels, located close to gastrin storage in the cell body, were detected in the G cells of the mouse [65]. However, the role of Piezo1 channels in mechanosensing is unclear. TRPV2 channels are expressed in most neuronal nitric oxide synthase-expressing myenteric neurons (inhibitory motor neurons) in the stomach and may play a role in mechanosensing during distension of the gastric wall [66]. 

### 3.2. Distension-Evoked Peristalsis

Peristalsis is a propagated series of contractions in the gastrointestinal tract. Propagating waves of contractions can be evoked in the small intestine in response to distension (e.g., from a bolus of ingested material) and contraction frequencies are dictated by the slow waves [67]. However, there is controversy over whether the propagating waves are peristaltic reflexes, at least in the mouse model [67,68]. The mechanisms for the peristaltic reflex may be different for the small and large intestines [69]. In the colon, distension (circumferential stretch) activates intrinsic neurons that generate ascending and descending pathways resulting in peristalsis, and these pathways are resistant to L-type Ca^2+^ channel blockers [69].

Serotonin release from enterochromaffin cells clearly plays a role in regulating gastrointestinal motility, and serotonin is released in response to cell membrane deformation (i.e., during contraction). However, the release of serotonin in response to distension in the small intestine is still controversial [70]. Serotonin release may be evoked by the contraction of smooth muscle rather than distension, as formerly thought [22,23]. Distension-evoked peristalsis in either the small intestine or colon does not require the mucosa or serotonin [71,72]. The depletion of serotonin in the enteric neurons of the colon did not affect peristalsis [73]. This suggests, also, that the enterochromaffin release of serotonin is not involved in distension-evoked peristalsis. 

In contrast to the mucosa, the removal of the circular muscle, but not the longitudinal muscle, prevents distension-evoked peristalsis [74]. Connection of the circular muscle to the myenteric plexus is necessary for distension-evoked signaling [74,75]. Thus, the mechanosensors that are responsible for mediating distension-evoked peristalsis most likely reside in the circular smooth muscle and/or the myenteric plexus. However, the molecule responsible for sensing distension is not known. Peizo1 channels have been detected in nitrergic and cholinergic neurons of the myenteric plexus [42]. However, the role of Piezo1 channels in distension-evoked peristalsis is unclear.

## 4. Dysregulation of Mechanosensing Mechanisms in Gastrointestinal Diseases

Mechanotransduction mechanisms are involved in a number of gastrointestinal diseases, including postoperative ileus, irritable bowel syndrome, ulcerative colitis and Crohn’s disease [76], and functional dyspepsia [77]. Mechanotransduction may even play a role in gastric tumorigenesis and metastasis, which involve Piezo1 channels [78,79]. Ileus occurs in trauma patients (up to 30% [80]), post-surgical patients (10–30% of abdominal surgery patients [81]), and critical care patients (20–50% of intensive care unit patients [82]), affecting a very large population of hospitalized patients. In the case of ileus, overstimulation of normal physiological processes during distension of the bowel, for instance, due to surgical manipulation, bowel trauma, or edema development during trauma, may initiate the downstream processes that eventually result in the development of ileus. While ileus eventually resolves in most patients, ileus can cause feeding intolerance, necessitating intravenous nutrition, and resulting in increased infectious complications, longer hospital and intensive care unit stays, and increased hospital readmission, all of which increase patient care costs [80]. In contrast to ileus, inflammatory bowel syndrome (IBS) may develop due to the dysregulation of mechanotransduction pathways. IBS is the most common digestive disease, affecting 2–6% of the population worldwide (based on the Rome IV criteria), and has considerable patient welfare and economic impacts [83]. Of note, despite the large number of publications focused on ileus and IBS, the drugs to treat both of these diseases are relatively ineffective. Furthermore, dysregulation of mechanotransductive pathways likely plays a role in other GI motility disorders, such as gastroparesis. However, the role of mechanotransduction in these diseases is highly under-investigated and, thus, very little information is available.

### 4.1. Ileus

Ileus is the slowing or cessation of intestinal motility. The animal models for ileus include a gut manipulation model and a resuscitation-induced edema model [55,84,85]. In both of these models, increased mechanical force in the small intestine, i.e., mechanical pressure or increased stretch in response to the development of intestinal edema, results in the inhibition of intestinal motility [85,86,87,88,89]. In fact, the strength of surgical trauma correlates with the degree of postoperative intestinal dysmotility, indicating that ileus is at least partially triggered by mechanosensors [55]. Interestingly, manipulation of one section of the intestine inhibits intestinal motility in other areas of the GI tract, indicating that systemic mechanisms also play a role [90]. 

Mechanotransduction in macrophages plays a significant role in the development of ileus. Macrophages interact with both enteric neurons, interstitial cells of Cajal, and smooth muscle cells to affect intestinal motility [91]. Intestinal manipulation activates resident macrophages in the intestinal wall, which secrete proinflammatory mediators, resulting in the recruitment of neutrophils; overall, the mechanically-induced inflammatory reaction slows intestinal motility [92,93]. As part of a mechanically active organ, cells in the intestinal wall regularly experience mechanical activity, such as cyclical stretch. Physiological cyclical stretch does not induce inflammatory mediator release in macrophages. However, pathological cyclical stretch, as the intestinal wall might experience during ileus or edema development, induced the release of inflammatory mediators from macrophages, including CXCL1 [56]. Mechanotransduction in intestinal smooth muscle cells also plays a role in the development of ileus. Similar to macrophages, increased cyclical stretch of primary intestinal smooth muscle cells, but not physiological cyclical stretch, increases p21-activated kinase activity and CXCL1 secretion, which are associated with decreased intestinal motility [56,87]. Interestingly, CXCL1 is the first of a panel of cytokines that increased in the media after the pathological stretch of activated THP-1 cells [56]. CXCL1 increased within 1 h of increased cyclical stretch, while IL-1β increased after 4 h of increased cyclical stretch. 

Although mechanotransduction clearly plays a role in the development of ileus, the mechanosensors involved in the development of ileus are not known. However, TRPV4 plays a significant role in mechanosensation in macrophages and enteric neurons [94]. The genetic deficiency of TRPM2, which is expressed in intestinal macrophages and enteric neurons, prevents the development of ileus (gut manipulation model), suggesting that TRPM2 channels may be involved in the mechanotransduction of ileus development [95]. Mechanotransduction plays a clear role in the development of ileus, and understanding the mechanotransduction pathway(s), including the mechanosensors that trigger the development of ileus, will facilitate the development of more effective drugs to prevent or treat ileus.

### 4.2. Irritable Bowel Syndrome

While ileus can be thought of as an overstimulation of mechanosensitive pathways, the dysregulation rather than overstimulation of mechanosensitive pathways may be involved in the development of IBS. IBS is characterized by abdominal pain, bloating, and altered bowel habits. Repeated distension of the colon in IBS patients causes increased colonic motility compared with healthy individuals [96]. Furthermore, colonic distension in IBS patients fails to inhibit small intestinal motility in IBS patients, suggesting that the mechanotransduction pathways in patients with IBS are dysregulated [96]. In addition to abnormal gastrointestinal motility, IBS is characterized by visceral hypersensitivity, which is the altered sensation of physiological stimuli [97]. 

As discussed in Section 2.1.1, serotonin plays an important role in mechanotransductive mechanisms in the gastrointestinal tract. Serotonin and tryptophan hydroxylase are reduced in the colonic mucosae of patients with IBS compared with the levels in healthy individuals [98]. The levels of serotonin transporter, responsible for serotonin reuptake, are also decreased in the colon of IBS patients [98]. An altered serotonin transporter is associated with both diarrhea and constipation [99]. 

Visceral pain is a hallmark of IBS. IBS patients display enhanced responses to distension of the colorectum [100]. Mechanosensitive ion channels, including Piezo2, TRPV4, TRPA1, and Nav channels, have been implicated in enhanced visceral pain in IBS [101]. Knockdown of Piezo2 channels increased visceral sensation [102]. The number of TRPV4-expressing nerve fibers is significantly higher in IBS patients, and inhibition or knockdown of TRPV4 reduces sensitivity to colorectal pain [103,104]. More nerve fibers expressing Nav1.7 (sodium voltage-gated channel, encoded by *SCN9A*) were detected in patients with idiopathic hypersensitivity than in healthy individuals, and blocking Nav1.8 prevented mechanically induced hyperalgesia in patients [54,105]. Mutations in mechanosensitive Nav1.5 channels (*SCN5A*), which are expressed in both smooth muscle cells and interstitial cells of Cajal, are found in 2% of IBS patients [106]. The G615E mutation of *SCN5A*, which encodes NAV1.5, induced the loss of mechanosensitivity [107].

## 5. Conclusions

Gastrointestinal function is highly dependent on sensing and responding to both physiological and pathological mechanical cues. Virtually all cell types in the gastrointestinal tract, including enteric neurons, enterochromaffin cells, smooth muscle cells, interstitial cells of Cajal, and macrophages, rely on mechanotransduction for normal function. Thus, it is not surprising that many gastrointestinal diseases, such as ileus and IBS, involve dysregulation or overstimulation of mechanical signals. Unfortunately, mechanotransduction, especially the molecular mechanisms for mechanosensing, is poorly understood. Both ileus and IBS lack effective treatments. Understanding the mechanotransduction mechanisms involved in these diseases will facilitate the identification of new drug targets to effectively treat these diseases.

## Figures and Tables

**Table 1:** Potential mechanosensors in the gastrointestinal tract.

Cell Type	Mechanosensor	Stimuli *	Effect	References
Enterochromaffin cells	Piezo2	Pressure; distension; luminal signals; shear stress	Ca^2+^ influx; serotonin release	[6,7,8]
Enteric neurons	TRPA1	Distension	Spike discharges	[9,10]
	BKCa	Stretch	Cell hyperpolarization	[11,12]
	K2P (TREK2 and TRAAK)	Shear stress; negative membrane pressure; stretch	Hyperpolarization; regulate resting membrane potential	[13]
	TRPV4	Distension	Spike discharges	[14]
Interstitial cells of Cajal	L-Type Ca channel	Shear stress; membrane tension; pressure	Increased inward current; faster activation	[15,16]
	Na_v_1.5	Stretch	Increases slow-wave frequency	[17]
Smooth muscle cells	L-Type Ca channel	Shear stress; membrane tension; pressure	Increased inward current; faster activation	[16,18]
	BK channels	Stretch	Regulation of excitability	[12]
	Na_v_1.5	Shear stress	Increased excitability	[19]
	K2P (TREK1)	Stretch	Hyperpolarization	[13,20]
Macrophages	TRPV4	Stretch	Spike discharges	[21]

* This includes published data in the gut. Other stimuli may also signal through the mechanosensor.

## Data Availability

Not applicable.

## References

[1] Mercado-Perez A., Beyder A. (2022). Gut feelings: Mechanosensing in the gastrointestinal tract. Nat. Rev. Gastroenterol. Hepatol..

[2] Bayliss W.M., Starling E.H. (1899). The movements and innervation of the small intestine. J. Physiol..

[3] Kim M., Heo G., Kim S.Y. (2022). Neural signalling of gut mechanosensation in ingestive and digestive processes. Nat. Rev. Neurosci..

[4] Joshi V., Strege P.R., Farrugia G., Beyder A. (2021). Mechanotransduction in gastrointestinal smooth muscle cells: Role of mechanosensitive ion channels. Am. J. Physiol. Gastrointest. Liver Physiol..

[5] Uray I.P., Uray K. (2021). Mechanotransduction at the Plasma Membrane-Cytoskeleton Interface. Int. J. Mol. Sci..

[6] Wang F., Knutson K., Alcaino C., Linden D.R., Gibbons S.J., Kashyap P., Grover M., Oeckler R., Gottlieb P.A., Li H.J. (2017). Mechanosensitive ion channel Piezo2 is important for enterochromaffin cell response to mechanical forces. J. Physiol..

[7] Alcaino C., Knutson K.R., Treichel A.J., Yildiz G., Strege P.R., Linden D.R., Li J.H., Leiter A.B., Szurszewski J.H., Farrugia G. (2018). A population of gut epithelial enterochromaffin cells is mechanosensitive and requires Piezo2 to convert force into serotonin release. Proc. Natl. Acad. Sci. USA.

[8] Treichel A.J., Finholm I., Knutson K.R., Alcaino C., Whiteman S.T., Brown M.R., Matveyenko A., Wegner A., Kacmaz H., Mercado-Perez A. (2022). Specialized Mechanosensory Epithelial Cells in Mouse Gut Intrinsic Tactile Sensitivity. Gastroenterology.

[9] Brierley S.M., Hughes P.A., Page A.J., Kwan K.Y., Martin C.M., O’Donnell T.A., Cooper N.J., Harrington A.M., Adam B., Liebregts T. (2009). The ion channel TRPA1 is required for normal mechanosensation and is modulated by algesic stimuli. Gastroenterology.

[10] Bielefeldt K., Davis B.M. (2008). Differential effects of ASIC3 and TRPV1 deletion on gastroesophageal sensation in mice. Am. J. Physiol. Gastrointest. Liver Physiol..

[11] Kefauver J.M., Ward A.B., Patapoutian A. (2020). Discoveries in structure and physiology of mechanically activated ion channels. Nature.

[12] Wang W., Huang H., Hou D., Liu P., Wei H., Fu X., Niu W. (2010). Mechanosensitivity of STREX-lacking BKCa channels in the colonic smooth muscle of the mouse. Am. J. Physiol. Gastrointest. Liver Physiol..

[13] Sanders K.M., Koh S.D. (2006). Two-pore-domain potassium channels in smooth muscles: New components of myogenic regulation. J. Physiol..

[14] Brierley S.M., Page A.J., Hughes P.A., Adam B., Liebregts T., Cooper N.J., Holtmann G., Liedtke W., Blackshaw L.A. (2008). Selective role for TRPV4 ion channels in visceral sensory pathways. Gastroenterology.

[15] Lyford G.L., Farrugia G. (2003). Ion channels in gastrointestinal smooth muscle and interstitial cells of Cajal. Curr. Opin. Pharmacol..

[16] Lyford G.L., Strege P.R., Shepard A., Ou Y., Ermilov L., Miller S.M., Gibbons S.J., Rae J.L., Szurszewski J.H., Farrugia G. (2002). alpha(1C) (Ca(V)1.2) L-type calcium channel mediates mechanosensitive calcium regulation. Am. J. Physiol. Cell Physiol..

[17] Strege P.R., Ou Y., Sha L., Rich A., Gibbons S.J., Szurszewski J.H., Sarr M.G., Farrugia G. (2003). Sodium current in human intestinal interstitial cells of Cajal. Am. J. Physiol. Gastrointest. Liver Physiol..

[18] Farrugia G., Holm A.N., Rich A., Sarr M.G., Szurszewski J.H., Rae J.L. (1999). A mechanosensitive calcium channel in human intestinal smooth muscle cells. Gastroenterology.

[19] Neshatian L., Strege P.R., Rhee P.L., Kraichely R.E., Mazzone A., Bernard C.E., Cima R.R., Larson D.W., Dozois E.J., Kline C.F. (2015). Ranolazine inhibits voltage-gated mechanosensitive sodium channels in human colon circular smooth muscle cells. Am. J. Physiol. Gastrointest. Liver Physiol..

[20] Koh S.D., Sanders K.M. (2001). Stretch-dependent potassium channels in murine colonic smooth muscle cells. J. Physiol..

[21] Goswami R., Merth M., Sharma S., Alharbi M.O., Aranda-Espinoza H., Zhu X., Rahaman S.O. (2017). TRPV4 calcium-permeable channel is a novel regulator of oxidized LDL-induced macrophage foam cell formation. Free Radic. Biol. Med..

[22] Bulbring E., Crema A. (1959). The release of 5-hydroxytryptamine in relation to pressure exerted on the intestinal mucosa. J. Physiol..

[23] Bertrand P.P. (2006). Real-time measurement of serotonin release and motility in guinea pig ileum. J. Physiol..

[24] Patel B.A., Bian X., Quaiserova-Mocko V., Galligan J.J., Swain G.M. (2007). In vitro continuous amperometric monitoring of 5-hydroxytryptamine release from enterochromaffin cells of the guinea pig ileum. Analyst.

[25] Linan-Rico A., Ochoa-Cortes F., Beyder A., Soghomonyan S., Zuleta-Alarcon A., Coppola V., Christofi F.L. (2016). Mechanosensory Signaling in Enterochromaffin Cells and 5-HT Release: Potential Implications for Gut Inflammation. Front. Neurosci..

[26] Gershon M.D., Tack J. (2007). The serotonin signaling system: From basic understanding to drug development for functional GI disorders. Gastroenterology.

[27] Heredia D.J., Dickson E.J., Bayguinov P.O., Hennig G.W., Smith T.K. (2009). Localized release of serotonin (5-hydroxytryptamine) by a fecal pellet regulates migrating motor complexes in murine colon. Gastroenterology.

[28] Heredia D.J., Gershon M.D., Koh S.D., Corrigan R.D., Okamoto T., Smith T.K. (2013). Important role of mucosal serotonin in colonic propulsion and peristaltic reflexes: In vitro analyses in mice lacking tryptophan hydroxylase 1. J. Physiol..

[29] Beyder A. (2018). In Pursuit of the Epithelial Mechanosensitivity Mechanisms. Front. Endocrinol. (Lausanne).

[30] Cooke H.J., Wunderlich J., Christofi F.L. (2003). “The force be with you”: ATP in gut mechanosensory transduction. News Physiol. Sci..

[31] Bertrand P.P., Kunze W.A., Furness J.B., Bornstein J.C. (2000). The terminals of myenteric intrinsic primary afferent neurons of the guinea-pig ileum are excited by 5-hydroxytryptamine acting at 5-hydroxytryptamine-3 receptors. Neuroscience.

[32] Ranade S.S., Woo S.H., Dubin A.E., Moshourab R.A., Wetzel C., Petrus M., Mathur J., Begay V., Coste B., Mainquist J. (2014). Piezo2 is the major transducer of mechanical forces for touch sensation in mice. Nature.

[33] Kugler E.M., Michel K., Zeller F., Demir I.E., Ceyhan G.O., Schemann M., Mazzuoli-Weber G. (2015). Mechanical stress activates neurites and somata of myenteric neurons. Front. Cell Neurosci..

[34] Mazzuoli G., Schemann M. (2012). Mechanosensitive enteric neurons in the myenteric plexus of the mouse intestine. PLoS ONE.

[35] Mazzuoli-Weber G., Schemann M. (2015). Mechanosensitive enteric neurons in the guinea pig gastric corpus. Front. Cell Neurosci..

[36] Wood J.D. (1970). Electrical activity from single neurons in Auerbach’s plexus. Am. J. Physiol..

[37] Mao Y., Wang B., Kunze W. (2006). Characterization of myenteric sensory neurons in the mouse small intestine. J. Neurophysiol..

[38] Mazzuoli G., Schemann M. (2009). Multifunctional rapidly adapting mechanosensitive enteric neurons (RAMEN) in the myenteric plexus of the guinea pig ileum. J. Physiol..

[39] Kugler E.M., Michel K., Kirchenbuchler D., Dreissen G., Csiszar A., Merkel R., Schemann M., Mazzuoli-Weber G. (2018). Sensitivity to Strain and Shear Stress of Isolated Mechanosensitive Enteric Neurons. Neuroscience.

[40] Mazzuoli-Weber G., Schemann M. (2015). Mechanosensitivity in the enteric nervous system. Front. Cell Neurosci..

[41] Spencer N.J., Smith C.B., Smith T.K. (2001). Role of muscle tone in peristalsis in guinea-pig small intestine. J. Physiol..

[42] Mazzuoli-Weber G., Kugler E.M., Buhler C.I., Kreutz F., Demir I.E., Ceyhan O.G., Zeller F., Schemann M. (2019). Piezo proteins: Incidence and abundance in the enteric nervous system. Is there a link with mechanosensitivity?. Cell Tissue Res..

[43] Li Z., Chalazonitis A., Huang Y.Y., Mann J.J., Margolis K.G., Yang Q.M., Kim D.O., Cote F., Mallet J., Gershon M.D. (2011). Essential roles of enteric neuronal serotonin in gastrointestinal motility and the development/survival of enteric dopaminergic neurons. J. Neurosci..

[44] Yadav V.K., Balaji S., Suresh P.S., Liu X.S., Lu X., Li Z., Guo X.E., Mann J.J., Balapure A.K., Gershon M.D. (2010). Pharmacological inhibition of gut-derived serotonin synthesis is a potential bone anabolic treatment for osteoporosis. Nat. Med..

[45] Wang Z.Y., Han Y.F., Huang X., Zhao P., Lu H.L., Kim Y.C., Xu W.X. (2010). Pacemaking activity is regulated by membrane stretch via the CICR pathway in cultured interstitial cells of Cajal from murine intestine. J. Biomech..

[46] Won K.J., Sanders K.M., Ward S.M. (2005). Interstitial cells of Cajal mediate mechanosensitive responses in the stomach. Proc. Natl. Acad. Sci. USA.

[47] Huizinga J.D., Thuneberg L., Kluppel M., Malysz J., Mikkelsen H.B., Bernstein A. (1995). W/kit gene required for interstitial cells of Cajal and for intestinal pacemaker activity. Nature.

[48] Kraichely R.E., Farrugia G. (2007). Mechanosensitive ion channels in interstitial cells of Cajal and smooth muscle of the gastrointestinal tract. Neurogastroenterol. Motil..

[49] Treichel A.J., Farrugia G., Beyder A. (2018). The touchy business of gastrointestinal (GI) mechanosensitivity. Brain Res..

[50] Beyder A., Farrugia G. (2012). Targeting ion channels for the treatment of gastrointestinal motility disorders. Therap. Adv. Gastroenterol..

[51] Kunze W.A., Clerc N., Bertrand P.P., Furness J.B. (1999). Contractile activity in intestinal muscle evokes action potential discharge in guinea-pig myenteric neurons. J. Physiol..

[52] Strege P.R., Holm A.N., Rich A., Miller S.M., Ou Y., Sarr M.G., Farrugia G. (2003). Cytoskeletal modulation of sodium current in human jejunal circular smooth muscle cells. Am. J. Physiol. Cell Physiol..

[53] Tsvilovskyy V.V., Zholos A.V., Aberle T., Philipp S.E., Dietrich A., Zhu M.X., Birnbaumer L., Freichel M., Flockerzi V. (2009). Deletion of TRPC4 and TRPC6 in mice impairs smooth muscle contraction and intestinal motility in vivo. Gastroenterology.

[54] De Schepper S., Stakenborg N., Matteoli G., Verheijden S., Boeckxstaens G.E. (2018). Muscularis macrophages: Key players in intestinal homeostasis and disease. Cell Immunol..

[55] Kalff J.C., Schraut W.H., Simmons R.L., Bauer A.J. (1998). Surgical manipulation of the gut elicits an intestinal muscularis inflammatory response resulting in postsurgical ileus. Ann. Surg..

[56] Docsa T., Bhattarai D., Sipos A., Wade C.E., Cox C.S., Uray K. (2020). CXCL1 is upregulated during the development of ileus resulting in decreased intestinal contractile activity. Neurogastroenterol. Motil..

[57] Kalff J.C., Turler A., Schwarz N.T., Schraut W.H., Lee K.K., Tweardy D.J., Billiar T.R., Simmons R.L., Bauer A.J. (2003). Intra-abdominal activation of a local inflammatory response within the human muscularis externa during laparotomy. Ann. Surg..

[58] Wehner S., Buchholz B.M., Schuchtrup S., Rocke A., Schaefer N., Lysson M., Hirner A., Kalff J.C. (2010). Mechanical strain and TLR4 synergistically induce cell-specific inflammatory gene expression in intestinal smooth muscle cells and peritoneal macrophages. Am. J. Physiol. Gastrointest. Liver Physiol..

[59] Jetten N., Verbruggen S., Gijbels M.J., Post M.J., De Winther M.P., Donners M.M. (2014). Anti-inflammatory M2, but not pro-inflammatory M1 macrophages promote angiogenesis in vivo. Angiogenesis.

[60] Ballotta V., Driessen-Mol A., Bouten C.V., Baaijens F.P. (2014). Strain-dependent modulation of macrophage polarization within scaffolds. Biomaterials.

[61] Atcha H., Jairaman A., Holt J.R., Meli V.S., Nagalla R.R., Veerasubramanian P.K., Brumm K.T., Lim H.E., Othy S., Cahalan M.D. (2021). Mechanically activated ion channel Piezo1 modulates macrophage polarization and stiffness sensing. Nat. Commun..

[62] McWhorter F.Y., Davis C.T., Liu W.F. (2015). Physical and mechanical regulation of macrophage phenotype and function. Cell Mol. Life Sci..

[63] Schubert M.L., Makhlouf G.M. (1993). Gastrin secretion induced by distention is mediated by gastric cholinergic and vasoactive intestinal peptide neurons in rats. Gastroenterology.

[64] Dixit D., Zarate N., Liu L.W., Boreham D.R., Huizinga J.D. (2006). Interstitial cells of Cajal and adaptive relaxation in the mouse stomach. Am. J. Physiol. Gastrointest. Liver Physiol..

[65] Lang K., Breer H., Frick C. (2018). Mechanosensitive ion channel Piezo1 is expressed in antral G cells of murine stomach. Cell Tissue Res..

[66] Mihara H., Suzuki N., Yamawaki H., Tominaga M., Sugiyama T. (2013). TRPV2 ion channels expressed in inhibitory motor neurons of gastric myenteric plexus contribute to gastric adaptive relaxation and gastric emptying in mice. Am. J. Physiol. Gastrointest. Liver Physiol..

[67] Seerden T.C., Lammers W.J., De Winter B.Y., De Man J.G., Pelckmans P.A. (2005). Spatiotemporal electrical and motility mapping of distension-induced propagating oscillations in the murine small intestine. Am. J. Physiol. Gastrointest. Liver Physiol..

[68] Huizinga J.D., McKay C.M., White E.J. (2006). The many facets of intestinal peristalsis. Am. J. Physiol. Gastrointest. Liver Physiol..

[69] Spencer N.J., Hennig G.W., Smith T.K. (2002). A rhythmic motor pattern activated by circumferential stretch in guinea-pig distal colon. J. Physiol..

[70] Liu Y.L., Chen Y., Fan W.T., Cao P., Yan J., Zhao X.Z., Dong W.G., Huang W.H. (2020). Mechanical Distension Induces Serotonin Release from Intestine as Revealed by Stretchable Electrochemical Sensing. Angew. Chem. Int. Ed. Engl..

[71] Spencer N.J., Nicholas S.J., Robinson L., Kyloh M., Flack N., Brookes S.J., Zagorodnyuk V.P., Keating D.J. (2011). Mechanisms underlying distension-evoked peristalsis in guinea pig distal colon: Is there a role for enterochromaffin cells?. Am. J. Physiol. Gastrointest. Liver Physiol..

[72] Tsuji S., Anglade P., Ozaki T., Sazi T., Yokoyama S. (1992). Peristaltic movement evoked in intestinal tube devoid of mucosa and submucosa. Jpn. J. Physiol..

[73] Sia T.C., Flack N., Robinson L., Kyloh M., Nicholas S.J., Brookes S.J., Wattchow D.A., Dinning P., Oliver J., Spencer N.J. (2013). Is serotonin in enteric nerves required for distension-evoked peristalsis and propulsion of content in guinea-pig distal colon?. Neuroscience.

[74] Spencer N.J., Dickson E.J., Hennig G.W., Smith T.K. (2006). Sensory elements within the circular muscle are essential for mechanotransduction of ongoing peristaltic reflex activity in guinea-pig distal colon. J. Physiol..

[75] Lomax A.E., Sharkey K.A., Bertrand P.P., Low A.M., Bornstein J.C., Furness J.B. (1999). Correlation of morphology, electrophysiology and chemistry of neurons in the myenteric plexus of the guinea-pig distal colon. J. Auton. Nerv. Syst..

[76] Moriggi M., Pastorelli L., Torretta E., Tontini G.E., Capitanio D., Bogetto S.F., Vecchi M., Gelfi C. (2017). Contribution of Extracellular Matrix and Signal Mechanotransduction to Epithelial Cell Damage in Inflammatory Bowel Disease Patients: A Proteomic Study. Proteomics.

[77] Page A.J., Li H. (2018). Meal-Sensing Signaling Pathways in Functional Dyspepsia. Front. Syst. Neurosci..

[78] Wang X., Cheng G., Miao Y., Qiu F., Bai L., Gao Z., Huang Y., Dong L., Niu X., Wang X. (2021). Piezo type mechanosensitive ion channel component 1 facilitates gastric cancer omentum metastasis. J. Cell Mol. Med..

[79] Zhang J., Zhou Y., Tang P.M.K., Cheng A.S.L., Yu J., To K.F., Kang W. (2019). Mechanotransduction and Cytoskeleton Remodeling Shaping YAP1 in Gastric Tumorigenesis. Int. J. Mol. Sci..

[80] Virani F.R., Peery T., Rivas O., Tomasek J., Huerta R., Wade C.E., Lee J., Holcomb J.B., Uray K. (2019). Incidence and Effects of Feeding Intolerance in Trauma Patients. Jpen. J. Parenter. Enteral. Nutr..

[81] Venara A., Neunlist M., Slim K., Barbieux J., Colas P.A., Hamy A., Meurette G. (2016). Postoperative ileus: Pathophysiology, incidence, and prevention. J. Visc. Surg..

[82] Caddell K.A., Martindale R., McClave S.A., Miller K. (2011). Can the intestinal dysmotility of critical illness be differentiated from postoperative ileus?. Curr. Gastroenterol. Rep..

[83] Sperber A.D. (2021). Epidemiology and Burden of Irritable Bowel Syndrome: An International Perspective. Gastroenterol. Clin. North Am..

[84] Cox C.S., Radhakrishnan R., Villarrubia L., Xue H., Uray K., Gill B.S., Stewart R.H., Laine G.A. (2008). Hypertonic saline modulation of intestinal tissue stress and fluid balance. Shock.

[85] Uray K.S., Laine G.A., Xue H., Allen S.J., Cox C.S. (2006). Intestinal edema decreases intestinal contractile activity via decreased myosin light chain phosphorylation. Crit. Care Med..

[86] Chu J., Miller C.T., Kislitsyna K., Laine G.A., Stewart R.H., Cox C.S., Uray K.S. (2012). Decreased myosin phosphatase target subunit 1(MYPT1) phosphorylation via attenuated rho kinase and zipper-interacting kinase activities in edematous intestinal smooth muscle. Neurogastroenterol. Motil..

[87] Chu J., Pham N.T., Olate N., Kislitsyna K., Day M.C., LeTourneau P.A., Kots A., Stewart R.H., Laine G.A., Cox C.S. (2013). Biphasic regulation of myosin light chain phosphorylation by p21-activated kinase modulates intestinal smooth muscle contractility. J. Biol. Chem..

[88] Uray K.S., Laine G.A., Xue H., Allen S.J., Cox C.S. (2007). Edema-induced intestinal dysfunction is mediated by STAT3 activation. Shock.

[89] Uray K.S., Wright Z., Kislitsyna K., Xue H., Cox C.S. (2010). Nuclear factor-kappaB activation by edema inhibits intestinal contractile activity. Crit. Care Med..

[90] Schwarz N.T., Kalff J.C., Turler A., Speidel N., Grandis J.R., Billiar T.R., Bauer A.J. (2004). Selective jejunal manipulation causes postoperative pan-enteric inflammation and dysmotility. Gastroenterology.

[91] Mikkelsen H.B. (2010). Interstitial cells of Cajal, macrophages and mast cells in the gut musculature: Morphology, distribution, spatial and possible functional interactions. J. Cell Mol. Med..

[92] Boeckxstaens G.E., de Jonge W.J. (2009). Neuroimmune mechanisms in postoperative ileus. Gut.

[93] Wehner S., Behrendt F.F., Lyutenski B.N., Lysson M., Bauer A.J., Hirner A., Kalff J.C. (2007). Inhibition of macrophage function prevents intestinal inflammation and postoperative ileus in rodents. Gut.

[94] Michalick L., Kuebler W.M. (2020). TRPV4-A Missing Link Between Mechanosensation and Immunity. Front. Immunol..

[95] Matsumoto K., Kawanaka H., Hori M., Kusamori K., Utsumi D., Tsukahara T., Amagase K., Horie S., Yamamoto A., Ozaki H. (2018). Role of transient receptor potential melastatin 2 in surgical inflammation and dysmotility in a mouse model of postoperative ileus. Am. J. Physiol. Gastrointest. Liver Physiol..

[96] Fukudo S., Kanazawa M., Kano M., Sagami Y., Endo Y., Utsumi A., Nomura T., Hongo M. (2002). Exaggerated motility of the descending colon with repetitive distention of the sigmoid colon in patients with irritable bowel syndrome. J. Gastroenterol..

[97] Ritchie J. (1973). Pain from distension of the pelvic colon by inflating a balloon in the irritable colon syndrome. Gut.

[98] Coates M.D., Mahoney C.R., Linden D.R., Sampson J.E., Chen J., Blaszyk H., Crowell M.D., Sharkey K.A., Gershon M.D., Mawe G.M. (2004). Molecular defects in mucosal serotonin content and decreased serotonin reuptake transporter in ulcerative colitis and irritable bowel syndrome. Gastroenterology.

[99] Spigset O. (1999). Adverse reactions of selective serotonin reuptake inhibitors: Reports from a spontaneous reporting system. Drug. Saf..

[100] Naliboff B.D., Munakata J., Fullerton S., Gracely R.H., Kodner A., Harraf F., Mayer E.A. (1997). Evidence for two distinct perceptual alterations in irritable bowel syndrome. Gut.

[101] Yang H., Hou C., Xiao W., Qiu Y. (2022). The role of mechanosensitive ion channels in the gastrointestinal tract. Front. Physiol..

[102] Yang J., Zhang J., Yang H., Li K., Lei X., Xu C. (2016). The potential role of Piezo2 in the mediation of visceral sensation. Neurosci. Lett..

[103] Balemans D., Boeckxstaens G.E., Talavera K., Wouters M.M. (2017). Transient receptor potential ion channel function in sensory transduction and cellular signaling cascades underlying visceral hypersensitivity. Am. J. Physiol. Gastrointest. Liver Physiol..

[104] Winston J., Shenoy M., Medley D., Naniwadekar A., Pasricha P.J. (2007). The vanilloid receptor initiates and maintains colonic hypersensitivity induced by neonatal colon irritation in rats. Gastroenterology.

[105] Yiangou Y., Facer P., Chessell I.P., Bountra C., Chan C., Fertleman C., Smith V., Anand P. (2007). Voltage-gated ion channel Nav1.7 innervation in patients with idiopathic rectal hypersensitivity and paroxysmal extreme pain disorder (familial rectal pain). Neurosci. Lett..

[106] Strege P.R., Mazzone A., Bernard C.E., Neshatian L., Gibbons S.J., Saito Y.A., Tester D.J., Calvert M.L., Mayer E.A., Chang L. (2018). Irritable bowel syndrome patients have SCN5A channelopathies that lead to decreased NaV1.5 current and mechanosensitivity. Am. J. Physiol. Gastrointest. Liver Physiol..

[107] Strege P.R., Mercado-Perez A., Mazzone A., Saito Y.A., Bernard C.E., Farrugia G., Beyder A. (2019). SCN5A mutation G615E results in NaV1.5 voltage-gated sodium channels with normal voltage-dependent function yet loss of mechanosensitivity. Channels.

